# Development of a multiplex real-time qRT-PCR for discriminating the predominant epidemic variant IBDV and very virulent IBDV

**DOI:** 10.3389/fvets.2025.1736613

**Published:** 2026-01-21

**Authors:** Ziwen Wu, Hangbo Yu, Guodong Wang, Dan Ling, Yulong Zhang, Runhang Liu, Erjing Ke, Suyan Wang, Yanping Zhang, Yongzhen Liu, Hongyu Cui, Yuntong Chen, Yulu Duan, Xianyun Liu, Yulong Gao, Xiaole Qi

**Affiliations:** 1 Avian Immunosuppressive Diseases Division, State Key Laboratory for Animal Disease Control and Prevention, Harbin Veterinary Research Institute, The Chinese Academy of Agricultural Sciences, Harbin, China; 2 World Organization for Animal Health (WOAH) Reference Laboratory for Infectious Bursal Disease, Harbin Veterinary Research Institute, The Chinese Academy of Agricultural Sciences, Harbin, China; 3 Heilongjiang Province Key Laboratory of Veterinary Immunology, Harbin Veterinary Research Institute, The Chinese Academy of Agricultural Sciences, Harbin, China; 4 Jiangsu Co-Innovation Center for the Prevention and Control of Important Animal Infectious Disease and Zoonosis, Yangzhou University, Yangzhou, China

**Keywords:** infectious bursal disease virus, non-var/vvIBDV, qRT-PCR, varIBDV, vvIBDV

## Abstract

Infectious bursal disease (IBD) is an important immunosuppressive disease of chicken caused by infectious bursal disease virus (IBDV). At present, the newly emerging novel variant IBDV (varIBDV) and the persistently prevalent very virulent IBDV (vvIBDV) are two major threats, while the non-var/vvIBDV, such as classic IBDV (cIBDV) and attenuated IBDV (attIBDV), also increases the complexity of clinical detection. In this study, a multiplex real-time quantitative fluorescence RT-PCR (qRT-PCR) was developed. Based on sequence analysis of different pathogenic IBDV strains, three probes with different fluorescent signals (FAM, VIC, CY5) and two pairs of primers were designed. Specifically, varIBDV exhibits three fluorescent signals (FAM, VIC, CY5), vvIBDV shows two signals (FAM, VIC), and non-var/vvIBDV displays one signal (FAM). The method possesses excellent specificity: no cross-reactivity was observed between different pathogenic IBDV types, nor with other common avian pathogens. This method has good reproducibility and high sensitivity, with a minimum detection limit of about 10 copies. Furthermore, in the detection of laboratory or clinical samples, the consistency rate of this method with the conventional sequencing analysis method reached 100%. In conclusion, this study developed for the first time a multiplex qRT-PCR that can universally detect IBDV and simultaneously distinguish between vvIBDV and varIBDV, which is of great significance for high-throughput emergency detection and comprehensive prevention and control of new IBDV epidemics.

## 1 Introduction

Infectious bursal disease (IBD) is an acute, highly contagious, immunosuppressive disease caused by infectious bursal disease virus (IBDV) (1), which mainly harms chicks and leads to significant economic losses in the global poultry industry. IBDV is an icosahedral stereosymmetric, non-enveloped, double-stranded RNA virus that belongs to the *Avibirnavirus* genus under the *Birnaviridae* family. The IBDV genome is composed of two segments (A and B). Segment A (3.2 kb) encompasses two partially overlapping open reading frames (ORFs): the upstream smaller ORF encodes the non-structural protein VP5 (2), while the downstream larger ORF encodes the polyprotein of VP2-VP4-VP3. This polyprotein undergoes autoproteolysis to produced capsid protein VP2, viral serine protease VP4, and scaffolding protein VP3 (3). Notably, VP2 possesses a hypervariable region (HVR), which plays a crucial role in cell-tropism, virulence, and antigenic variation of IBDV (4–8). Segment B (2.8 kb) encoding the RNA-dependent RNA polymerase VP1, which plays a key role in the transcription and replication of viruses (9).

Since the first identification of IBDV in Gumboro, United States in 1957 (10), this virus has undergone numerous mutations and recombinations resulting in various pathotypes including classic IBDV (cIBDV), variant IBDV (varIBDV), and very virulent IBDV (vvIBDV) (11, 12). To prevent IBD, with blind-passage or reverse genetics, and the attenuated IBDV (attIBDV) from wild IBDV was developed as vaccines. Since 1989, vvIBDV has become one of the main threats facing the chicken industry with its high mortality and high transmission speed. In recent years, with the emergence and prevalence of novel varIBDV, the chicken industry is facing more complex challenges (13). The novel varIBDV exhibits significant differences in antigenicity compared to previous strains, resulting in existing vaccines being unable to provide complete immune protection against IBD (14, 15). It is precisely for these reasons that in many countries of Asian, African, and South American, the newly emerging varIBDV and persistently circulating vvIBDV are the two predominant epidemic strains endangering the poultry industry (16–19).

RT-PCR is a commonly used method for detecting IBDV, but it cannot directly identify different prevalent strains. Currently, the identification of varIBDV and vvIBDV can only rely on sequencing analysis, which is time-consuming, laborious, and expensive, and requires expert technicians. This study developed for the first time a multiplex real-time fluorescent quantitative RT-PCR (qRT-qPCR) that can universally detect IBDV and simultaneously distinguish between varIBDV and vvIBDV, which is of great significance for high-throughput emergency detection and comprehensive prevention and control of new IBDV epidemics.

## 2 Materials and methods

### 2.1 Primes and probes

**Figure 1:**
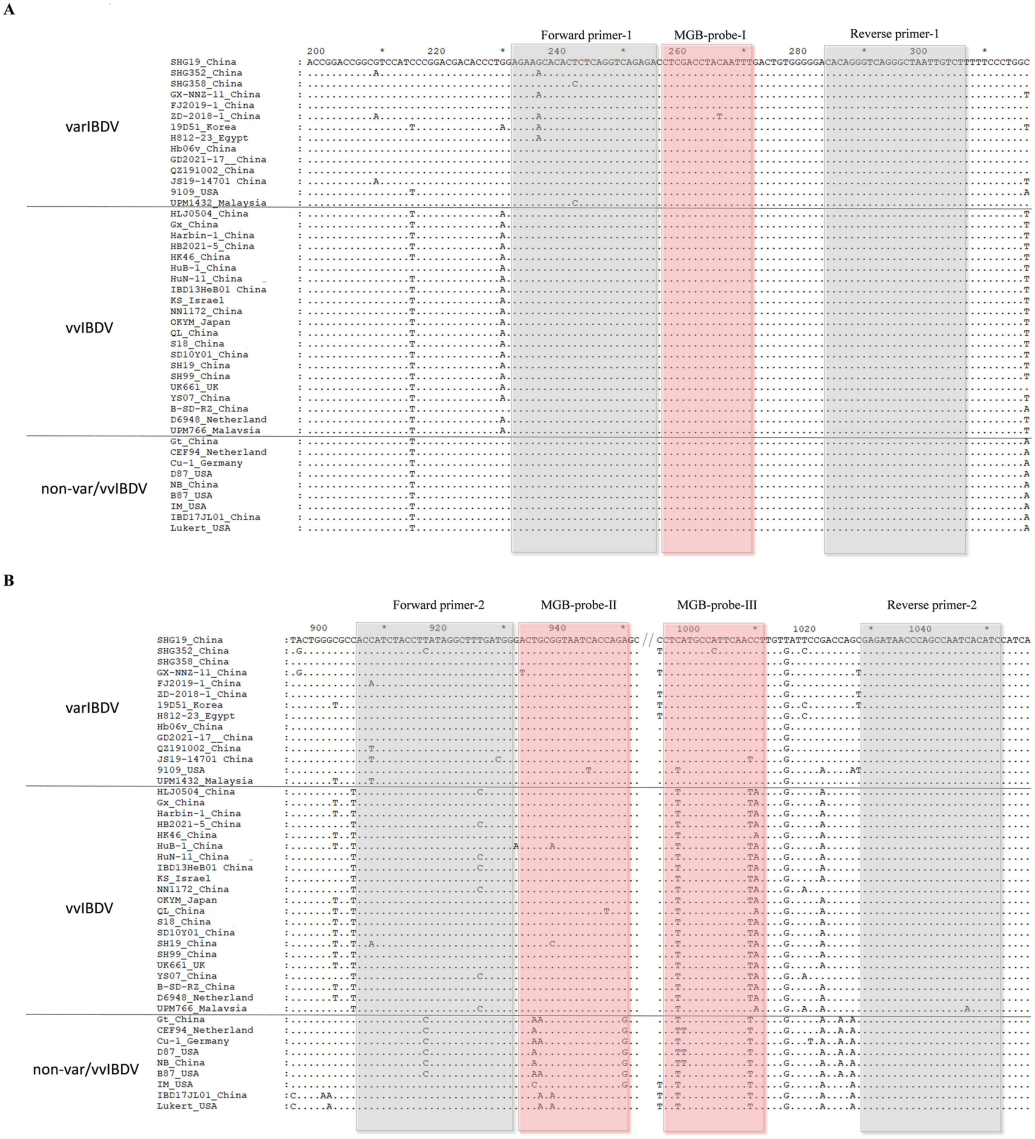
Nucleotide sequence alignment of the multiplex qRT-PCR amplification region among different pathotypes of IBDV. **(A)** Sequence fragment targeted by forward primer-1, reverse primer-1, and MGB-probe I. **(B)** Sequence fragment targeted by forward primer-2, reverse primer-2, MGB-probe II, and MGB-probe III. The position of amino acids is determined based on VP2 of varIBDV SHG19 strain (GenBank no. MH879092). Identical residues in aligned sequences are indicated by dots, and differences were indicated by single letters. Var, variant strain; vv, very virulent strain; non-var/vvIBDV, other IBDV strains besides var. and vv.

The VP2 gene sequences of different pathogenic IBDV strains from GenBank database^1^ were analyzed using the Megalign software (DNAStar) and GENEDOC software. Then, using Prime Express 3 software, two pairs of primers and three probes (Figure 1) targeting the VP2 gene of IBDV were designed for multiplex qRT-PCR to discriminate varIBDV, vvIBDV, and non-var/vvIBDV. The probe design followed a systematic SNP selection workflow: prioritizing key sites for strain discrimination, evaluating sequence conservation and flanking regions, and ensuring efficient allelic discrimination through central mismatch design. BLAST on the National Center for Biotechnology Information server^2^ was used to further confirm the specificity of the probes and primers. Probes and primers were synthesized by Sangon Biotech (Shanghai) Co., Ltd.

### 2.2 Virus strains and clinical samples

The varIBDV representative strains of varIBDV SHG19 (GenBank accession number MN393076), vvIBDV HLJ-0504 (GQ451330), cIBDV IBD17JL01 (MN604241.1), and attIBDV Gt (DQ403248) were identified by the Avian Immunosuppressive Disease Division, Harbin Veterinary Research Institute (HVRI), the Chinese Academy of Agricultural Sciences (CAAS) (hereinafter referred to as “our lab”) (13, 20–22). Avian influenza virus (AIV), Infectious bronchitis virus (IBV), Newcastle disease virus (NDV), Reticuloendotheliosis virus (REV), Fowl adenovirus serotype 4 (FAdV-4), Avian reovirus (ARV), Marek’s disease virus (MDV), Avian leukosis virus subgroup J (ALV-J), ALV subgroup K (ALV-K), *Mycoplasma gallisepticum* (*M. gallisepticum*), and *Mycoplasma synoviae* (*M. synoviae*) were also provided by HVRI and used to evaluate the specificity of IBDV multiplex qRT-PCR.

### 2.3 Virus RNA extraction and reverse transcription

Virus RNA was extracted using the FastPure Viral DNA/RNA Mini Kit (Vazyme, Nanjing, China) according to the manufacturer’s instructions. Then, the reverse transcription was performed using HiScript II Q RT SuperMix (Vazyme, Nanjing, China).

### 2.4 Construction of plasmid standards

To construct three recombinant plasmid standards representing different types of pathogenic strains, the gene fragments of VP2 from SHG19 strain (varIBDV), HLJ-0504 strain (vvIBDV), and Gt strain (non-var/vvIBDV) were amplified by RT-PCR using primers SHG19-F/SHG19-R, HLJ-0504-F/HLJ-0504-R, and Gt-F/ Gt-R (Table 1). The PCR products were ligated into the pMD18-T vector to obtain three plasmid standards: pMD18-T-SHG19, pMD18-T-HLJ-0504, and pMD18-T-Gt. The copy numbers of the plasmids were calculated using the following formula: copies/μL = [(plasmid concentration (ng/μL) × 6.02 × 10^23^)] / [(plasmid length (bp) × 1 × 10^9^ × 660 dalton/bp)] (23).

**Table 1:** Primers and probes.

Primers and probes	Sequences (5′ to 3′)	Position in VP2
Forward primer-1	AGAAGCACACTCTCAGGTCAGAGA	bp 231–254
Reverse primer-1	ACAATTAGCCCTGACCCTGTGT	bp 282–303
MGB-probe-I	FAM-CTCGACCTACAATTT-MGB	bp 256–270
Forward primer-2	ACCATCTACCTTATAGGCTTTGATGG	bp 908–933
Reverse primer-2	GATGTGATTGGCTGGGTTATCTC	bp 1,028–1,050
MGB-probe-II	VIC-ACTGCGGTAATCACCAGA-MGB	bp 935–952
MGB-probe-III	CY5-CTCATGCCATTCAACCT-MGB	bp 995–1,011
SHG19-F	GCCTTCTGATGCCAACAACCG	bp 180–200
SHG19-R	GGAGGTCACTATCTCCAATTT	bp 1,055–1,075
HLJ-0504-F	GCCTTCTGATGCCAACAACCG	bp 180–200
HLJ-0504-R	GGAGGTAACTATCTCCAGTTT	bp 1,055–1,075
Gt-F	GCCTTCTGATGCCAACAACCG	bp 180–200
Gt-R	GGAGGTCACTATCTCCAGTTT	bp 1,055–1,075

### 2.5 Real-time quantitative PCR

The multiple real-time qRT-PCR was developed to discriminate varIBDV, vvIBDV, and non-var/vvIBDV. The amplification reaction system was performed in QuantStudio 5 Real-Time PCR System (Applied Biosystems, United States). The total volume of the multiple real-time qRT-PCR reaction was 20 μL, consisting of 10 μL Premix EX Taq (Takara), 0.5 μL of forward primer-1, reverse primer-1, forward primer-2 and reverse primer-2 (10 μM), 0.5 μL of probe-I, probe-II and probe-III (10 μM), 2 μL template cDNA, and 4.5 μL RNase-free water. The reaction conditions were: 95 °C for 30s; 40 cycles of 95 °C for 3 s and 60 °C for 30s. After each cycle, the quantitative PCR instrument records the increase in fluorescence signal through an optical system, and the data including Ct value was calculated and analyzed through QuantStudio Real-Time PCR Systems.

### 2.6 Specificity of the qRT-PCR

To evaluate the specificity of this qRT-PCR, the DNA or cDNA of 11 other avian pathogens, including AIV (H9-GX11583 strain), IBV (H120), NDV (Lasota), REV (HLJR0901), FAdV-4 (HLJDAd15), ARV (ARV-HLJ21-1690401), MDV (LMS), ALV-J (HPRS103), ALV-K (JS15SG01), *M. gallisepticum* (R), and *M. synoviae* (WVU1853) were used as templates, with water as the negative control and SHG19 strain as the positive to perform the qRT-PCR.

### 2.7 Sensitivity of the qRT-PCR

To assess the sensitivity of this qRT-PCR, the real-time fluorescence quantitative analysis was conducted using the recombinant plasmid standards (pMD18-T-SHG19, pMD18-T-HLJ-0504, and pMD18-T-Gt) with different dilution gradients (10^7^ copies/μL to 10^1^ copies/μL). The standard curves and detection efficiency were automatically calculated and plotted. Furthermore, different strains of IBDV were used to further determine the sensitivity of qRT-PCR. Dilute the representative strains of each type by 10-fold ratio: SHG19 (varIBDV, 11,748 copies/μL), HLJ-0504 (vvIBDV, 15,840 copies/μL), and Gt (non-var/vvIBDV, 80,000 copies/μL). And the sensitivity of this qRT-PCR was detected as mentioned above.

### 2.8 Laboratory samples detection

Divide 9 four-week-old specific pathogen-free (SPF) chickens into three groups (A, B, and C), with 3 chickens in each group. Groups A and B were, respectively, inoculated with the varIBDV SHG19 and vvIBDV HLJ-0504, administering 100 μL (10 BID/mL) via nasal and ocular routes for each chicken. Group C served as the blank control group. Among them, one chicken in Group B died at 3 days post-inoculation (dpi) and two at 4 dpi. At 7 dpi, all surviving chickens were euthanized using high-concentration carbon dioxide inhalation, and all the bursa tissues were collected for analysis. Additionally, the attIBDV Gt and cIBDV IBD17JL01 were, respectively, inoculated into DF-1 cells, with cell suspension being collected 48 h post-inoculation. The samples of these four IBDV strains were used to evaluate this multiplex qRT-PCR.

### 2.9 Clinical samples detection

A total of 42 IBDV-positive clinical samples of bursa from Liaoning, Hebei, Shandong, Henan, Fujian, and Guangdong provinces of China in 2023–2024 were sent to our lab for IBDV detection. These clinical bursa samples were detected by the multiplex qRT-PCR. Meanwhile, the RT-PCR and conventional sequencing analysis was also performed as described previously (24).

### 2.10 Statistical analysis

All data analyses were performed using Prism software 10 (GraphPad Software, Inc.).

## 3 Results

### 3.1 Feasibility of the qRT-PCR

The main prevalent strains of IBDV in China can be divided into three types: varIBDV, vvIBDV, and non-var/vvIBDV (attIBDV and cIBDV). Through sequence alignment analysis of various pathogenic IBDV strains, it was found that the nucleotide sequence of bp 231–303 in VP2 is conserved across all types of IBDV strains. So, targeting this region, we designed the probe I (labeled with the FAM fluorescent dye) and the corresponding forward and reverse primers (Figure 1A) was designed for universal detection of IBDV. In addition, in the region of bp 908–1,050 of VP2, probe II (labeled with the VIC fluorescent dye), probe III (labeled with the CY5 fluorescent dye), and the corresponding forward and reverse primers were designed. Within the selected probe II region, varIBDV/vvIBDV exhibits 2–3 single nucleotide polymorphisms (SNPs) compared to non-var/vvIBDV, enabling differentiation varIBDV/vvIBDV from non-var/vvIBDV. Meanwhile, within the probe III region, varIBDV display 2–3 SNPs relative to vvIBDV and non-var/vvIBDV, allowing for the differentiation of varIBDV by probe III (Figure 1B).

**Figure 2:**
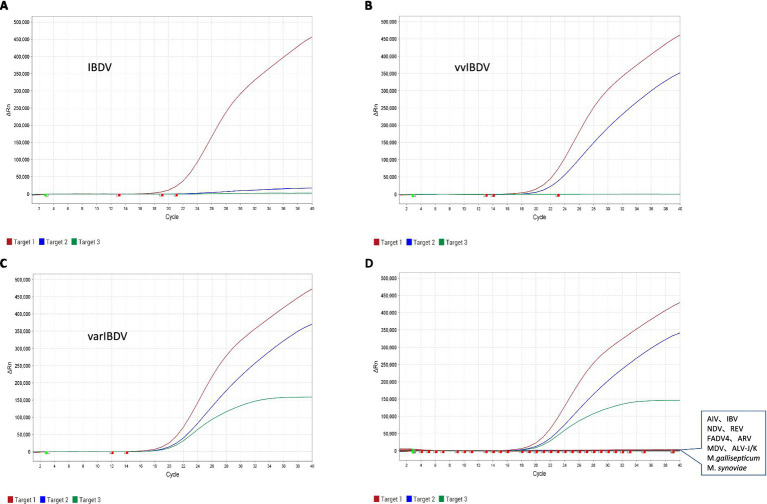
Specificity of the multiplex qRT-PCR. **(A–C)** Specific detection and amplification curve of the standard plasmid of non-var/vvIBDV **(A)**, vvIBDV **(B)**, and varIBDV **(C)**. **(D)** Specific detection and amplification curve of varIBDV (exhibits FAM, VIC, and CY5 fluorescent signals) and other non-IBDV samples including AIV, IBV, NDV, REV, FAdV-4, ARV, MDV, ALV-J, ALV-K, *M. gallisepticum*, *M. synoviae*, and ddH_2_O (no fluorescent signals).

Based on these probes and primers, we developed the multiple real-time qRT-PCR to distinguish and quantify varIBDV, vvIBDV, and non-var/vvIBDV. The test results of recombinant plasmid standards demonstrated that non-var/vvIBDV could be only detected in the FAM channel, indicating binding to probe I but not to probes II or III (Figure 2A); vvIBDV was recognized in the FAM and VIC channels, suggesting binding to probes I and II but not to probe III (Figure 2B); varIBDV could be detected in the FAM, VIC, and CY5 channels, indicating binding to probes I, II, and III (Figure 2C). In a word, for this IBDV multiple real-time qRT-PCR developed in this study, one signal (FAM) represents IBDV but non-var/vvIBDV, two signals (FAM/VIC) represent vvIBDV, three signals (FAM /VIC/CY5) represent varIBDV (Figure 2).

### 3.2 Specificity of the qRT-PCR

With this multiplex qRT-PCR of IBDV, only the positive control of IBDV was positive; all the non-IBDV samples, including AIV, IBV, NDV, REV, FAdV-4, ARV, MDV, ALV-J/K, *M. gallisepticum*, and *M. synoviae*, showed negative (Figure 2D). These results indicate that the multiplex qRT-PCR of IBDV exhibits good specificity.

### 3.3 Sensitivity of the qRT-PCR

**Figure 3:**
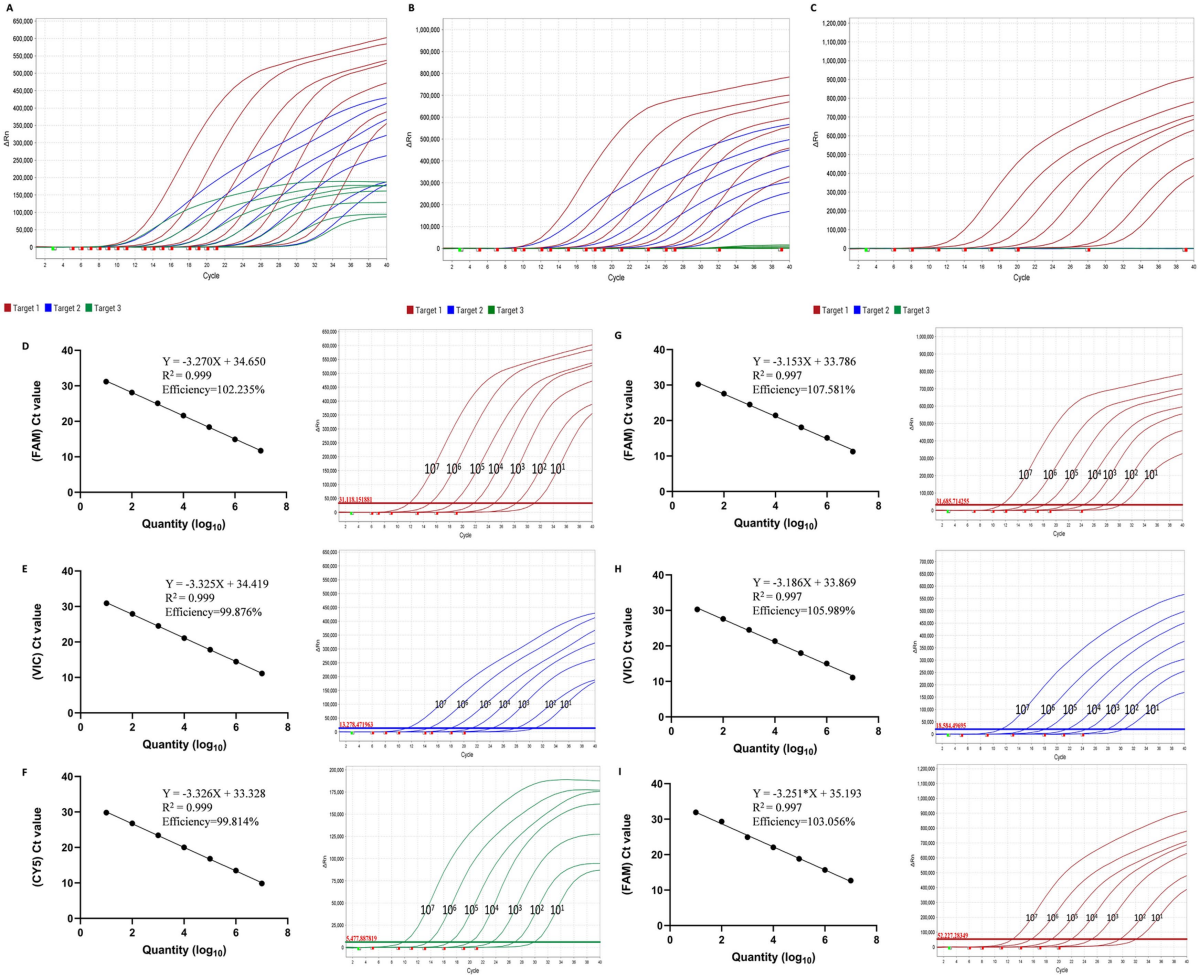
The plasmid standards sensitivity and standard curves of the multiplex RT-qPCR. **(A–C)** The sensitivity analysis of the 10-fold dilutions (10^7^ to 10^1^ copies/μL) of the standard plasmids of varIBDV **(A)**, vvIBDV **(B)**, and non-var/vvIBDV **(C)**. **(D–F)** The standard curve of the 10-fold dilutions (10^7^ to 10^1^ copies/μL) of the standard plasmids of varIBDV in channel FAM **(D)**, VIC **(E)**, and CY5 **(F)**. **(G,H)** The standard curve of the 10-fold dilutions (10^7^ to 10^1^ copies/μL) of the standard plasmids of vvIBDV in channel FAM **(G)** and VIC **(H)**. **(I)** The standard curve of the 10-fold dilutions (10^7^ to 10^1^ copies/μL) of the standard plasmids of non-var/vvIBDV in channel CY5 **(I)**.

The detection results of the recombinant plasmid standards of varIBDV (pMD18-T-SHG19) (Figure 3A), vvIBDV (pMD18-T-HLJ-0504) (Figure 3B), and non-var/vvIBDV (pMD18-T-Gt) (Figure 3C) at seven concentration gradients (10^7^–10^1^ copies/μL) showed positive, with each dilution tested in triplicate. For the pMD18-T-SHG19 standard plasmid, in the FAM fluorescence channel, the correlation coefficient R^2^ and amplification efficiency E of the equation were 0.999 and 102.235% (Figure 3D); in the VIC channel, the correlation coefficient R^2^ and amplification efficiency E of the equation were 0.999 and 99.876% (Figure 3E); and in the CY5 channel, the correlation coefficient R^2^ and amplification efficiency E of the equation were 0.999 and 99.814% (Figure 3F), respectively. For pMD18-T-HLJ-0504, in the FAM channel, the correlation coefficient R^2^ and amplification efficiency E of the equation were 0.997 and 107.581% (Figure 3G), respectively; in the VIC channel, the correlation coefficient R2 and amplification efficiency E of the equation were 0.997 and 105.989% (Figure 3H), respectively; for pMD18-T-Gt, in the FAM channel, the correlation coefficient R2 and amplification efficiency E of the equation were 0.997 and 103.056% (Figure 3I), respectively.

**Figure 4:**
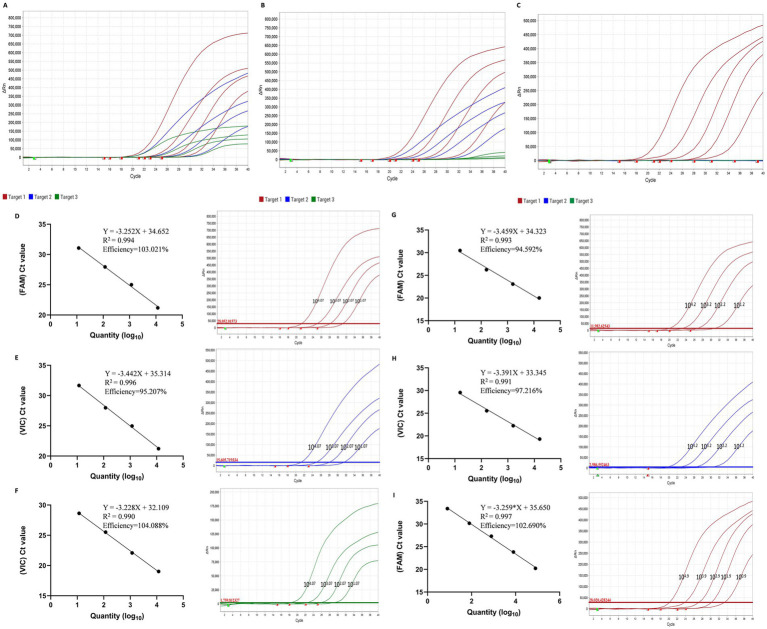
The virus sensitivity and standard curves of the multiplex RT-qPCR. **(A–C)** Sensitivity analysis of 10-fold serial dilutions for varIBDV SHG19 strain (10^4.07^ to 10^1.07^ copies/μL) **(A)**, vvIBDV HLJ0504 strain (10^4.2^ to 10^1.2^ copies/μL) **(B)**, and non-var/vvIBDV Gt strain (10^4.9^–10^0.9^ copies/μL) **(C)**. **(D–F)** The standard curve of the 10-fold dilutions (10^4.07^ to 10^1.07^ copies/μL) of the varIBDV strain in channel FAM **(D)**, VIC **(E)**, and CY5 **(F)**. **(G,H)** The standard curve of the 10-fold dilutions (10^4.2^ to 10^1.2^ copies/μL) of the vvIBDV strain in channel FAM **(G)** and VIC **(H)**. **(I)** The standard curve of the 10-fold dilutions (10^4.9^–10^0.9^ copies/μL) of non-var/vvIBDV strain in channel CY5.

For virus sample detection, varIBDV SHG19, vvIBDV HLJ-0504 and non-var/vvIBDV Gt were assayed at 4 (10^4.07^ to 10^1.07^ copies/μL) (Figure 4A), 4 (10^4.2^ to 10^1.2^ copies/μL) (Figure 4B), and 5 dilution gradients (10^4.9^ to 10^0.9^ copies/μL) (Figure 4C). Each gradient was tested in triplicate, and all dilution points tested positive. For the varIBDV SHG19, in the FAM fluorescence channel, the correlation coefficient R2 and amplification efficiency E of the equation were 0.994 and 103.021% (Figure 4D); in the VIC channel, the correlation coefficient R2 and amplification efficiency E of the equation were 0.996 and 95.207% (Figure 4E); and in the CY5 channel, the correlation coefficient R2 and amplification efficiency E of the equation were 0.990 and 104.088% (Figure 4F), respectively. For vvIBDV HLJ-0504, in the FAM channel, the correlation coefficient R2 and amplification efficiency E of the equation were 0.993 and 94.592% (Figure 4G), respectively; in the VIC channel, the correlation coefficient R2 and amplification efficiency E of the equation were 0.991 and 97.216% (Figure 4H), respectively; for non-var/vvIBDV Gt, in the FAM channel, the correlation coefficient R2 and amplification efficiency E of the equation were 0.997 and 102.690% (Figure 4I), respectively. These results indicate that the multiplex qRT-PCR of IBDV has satisfactory repeatability and sensitivity.

### 3.4 Laboratory samples detection

According to the results in Table 2, the varIBDV SHG19-infected samples were positive with three signals (FAM/VIC/CY5); the vvIBDV HLJ-0504-infected samples were positive with two signals (FAM/VIC); the non-var/vvIBDV IBD17JL01-infected and Gt-infected samples were positive with one signal (FAM); and the samples in NC group were negative.

**Table 2:** Detection of laboratory samples infected by different strains of IBDV.

Samples		qRT-PCR		
		FAM (Ct)	VIC (Ct)	CY5 (Ct)
SHG19 (varIBDV)	1	22.05	22.22	21.27
	2	22.48	22.73	21.69
	3	21.86	21.96	21.07
HLJ-0504 (vvIBDV)	1	23.79	24.15	Negative
	2	23.88	24.71	Negative
	3	23.87	24.26	Negative
Gt (attIBDV)	1	25.50	Negative	Negative
	2	25.32	Negative	Negative
	3	25.02	Negative	Negative
IBD17JL01 (cIBDV)	1	23.05	Negative	Negative
	2	23.90	Negative	Negative
	3	24.21	Negative	Negative
NC	1	Negative	Negative	Negative
	2	Negative	Negative	Negative
	3	Negative	Negative	Negative

### 3.5 Clinical sample detection

A total of 42 IBDV-positive clinical samples of bursa were detected by both multiplex qRT-PCR and conventional sequencing analysis. The results showed that 27 were positive for varIBDV, 8 were positive for vvIBDV, and 7 were positive for non-var/vvIBDV, which was consistent with the results of conventional sequencing analysis (Table 3).

**Table 3:** Clinical samples detection using the qRT-PCR.

Sample no.	qRT-PCR				VP2-HVR sequencing			Sample year
	FAM (Ct)	VIC (Ct)	CY5 (Ct)	Result	Phenotype	GenBank accession number	Genotype	
1	28.03	29.77	28.77	varIBDV	varIBDV	PQ673688	A2	2023
2	27.66	29.03	27.82	varIBDV	varIBDV	PQ673689	A2	2023
3	24.94	26.87	25.72	varIBDV	varIBDV	PQ673690	A2	2023
4	26.33	27.79	26.88	varIBDV	varIBDV	PQ673691	A2	2023
5	29.69	30.96	29.69	varIBDV	varIBDV	PQ673692	A2	2023
6	29.83	30.77	29.48	varIBDV	varIBDV	PQ673693	A2	2023
7	20.55	21.26	19.93	varIBDV	varIBDV	PQ673694	A2	2023
8	28.73	32.03	31.47	varIBDV	varIBDV	PQ673695	A2	2023
9	19.58	20.17	19.16	varIBDV	varIBDV	PQ673696	A2	2023
10	17.47	18.23	18.02	varIBDV	varIBDV	PQ673697	A2	2023
11	20.61	21.55	20.78	varIBDV	varIBDV	PQ673699	A2	2023
12	18.31	18.48	18.15	varIBDV	varIBDV	PQ673700	A2	2023
13	20.32	21.09	19.66	varIBDV	varIBDV	PQ673701	A2	2023
14	20.59	24.63	23.38	varIBDV	varIBDV	PQ673702	A2	2023
15	20.63	22.03	20.90	varIBDV	varIBDV	PQ673703	A2	2023
16	29.80	31.06	30.25	varIBDV	varIBDV	PQ673724	A2	2024
17	27.53	28.74	27.70	varIBDV	varIBDV	PQ673706	A2	2023
18	23.76	25.50	24.20	varIBDV	varIBDV	PQ673729	A2	2024
19	21.64	23.47	22.31	varIBDV	varIBDV	PQ673735	A2	2024
20	26.02	27.40	26.28	varIBDV	varIBDV	PQ673737	A2	2024
21	21.54	22.93	21.78	varIBDV	varIBDV	PQ673739	A2	2024
22	19.52	20.53	19.27	varIBDV	varIBDV	PQ673741	A2	2024
23	21.61	23.09	21.97	varIBDV	varIBDV	PQ673743	A2	2024
24	23.66	22.83	23.75	varIBDV	varIBDV	PQ673746	A2	2024
25	23.98	22.89	23.63	varIBDV	varIBDV	PQ673748	A2	2024
26	24.14	22.98	23.80	varIBDV	varIBDV	PQ673751	A2	2024
27	24.89	24.00	24.73	varIBDV	varIBDV	PQ673752	A2	2024
28	19.11	19.27	Negative	vvIBDV	vvIBDV	PQ673730	A3	2024
29	20.58	20.64	Negative	vvIBDV	vvIBDV	PQ673731	A3	2024
30	27.19	26.86	Negative	vvIBDV	vvIBDV	PQ673732	A3	2024
31	21.56	21.27	Negative	vvIBDV	vvIBDV	PQ673733	A3	2024
32	29.00	29.10	Negative	vvIBDV	vvIBDV	PQ673768	A3	2024
33	25.61	25.97	Negative	vvIBDV	vvIBDV	PQ673769	A3	2024
34	30.90	31.98	Negative	vvIBDV	vvIBDV	PQ673770	A3	2024
35	15.96	15.26	Negative	vvIBDV	vvIBDV	PQ673773	A3	2024
36	16.79	Negative	Negative	Non-varIBDV/vvIBDV	Non-varIBDV/vvIBDV	PQ673698	A1	2023
37	27.67	Negative	Negative	Non-varIBDV/vvIBDV	Non-varIBDV/vvIBDV	PQ673760	A8	2024
38	28.66	Negative	Negative	Non-varIBDV/vvIBDV	Non-varIBDV/vvIBDV	PQ673761	A8	2024
39	29.01	Negative	Negative	Non-varIBDV/vvIBDV	Non-varIBDV/vvIBDV	PQ673762	A8	2024
40	28.64	Negative	Negative	Non-varIBDV/vvIBDV	Non-varIBDV/vvIBDV	PQ673763	A8	2024
41	19.56	Negative	Negative	Non-varIBDV/vvIBDV	Non-varIBDV/vvIBDV	PQ673764	A8	2024
42	20.14	Negative	Negative	Non-varIBDV/vvIBDV	Non-varIBDV/vvIBDV	PQ673771	A1	2023

## 4 Discussion

Currently, multiple strains of IBDV coexist, and the clinically pathogenic strains mainly include vvIBDV, varIBDV, and cIBDV. Among them, the newly emerging varIBDV and persistently circulating vvIBDV are the two predominant epidemic strains endangering the poultry industry in many countries, including China (18, 25, 26). In some chicken farms, the harm of cIBDV also cannot be ignored. In addition, as a widely used vaccine, attIBDV is often detected in farms (27). Once the disease occurs in the chicken farm, quickly and accurately identifying the prevalent strain and taking matching measures is the top priority for efficient prevention and control of IBD. So, IBDV identification and detection technology is urgently needed.

From the perspective of pathogenic characteristics, the mortality of vvIBDV is relatively high, but in immunized chicken flocks, vvIBDV infection sometimes presents atypical IBD symptoms with low mortality, mainly manifested as severe lesion of bursa (28). The mortality rate of cIBDV is relatively low, and varIBDV does not directly kill chickens. Their main autopsy symptom is also typical bursa injury (15). The similarity of symptoms makes it difficult to achieve initial clinical detection of IBD. Farmers often send samples of suspected diseased chicken to the laboratory for testing. The conventional RT-PCR detection results are no longer able to meet the testing needs of farmers. When RT-PCR detected positive results for IBDV, they were eager to know what pathogenic types these strains were. The genetic characteristics of IBDV are closely related to its pathogenic type, and sequence analysis based on the VP2 gene is often used to determine the pathogenic type of IBDV (29–31). Although the sequencing analysis method is very accurate, for the demand of rapid clinical detection, it is time-consuming, laborious, and expensive, and requires expert technicians. The conventional sequencing method requires multiple steps (RT-PCR, agarose gel nucleic acid electrophoresis, PCR product purification, sequence analysis and interpretation), over a few days with limited throughput. From an economic perspective, the single sample cost of qRT-PCR is usually more than 20 times higher than conventional sequencing method. Moreover, when the RT-PCR production band is weak, sequencing results cannot be obtained. Therefore, it is urgent to develop a rapid identification and detection technology for IBDV, which involves determining whether it is positive for IBDV and identifying which dominant epidemic strain (varIBDV or vvIBDV) it is.

RT-PCR-restriction fragment length polymorphism (RT-PCR-RFLP) requires restriction enzyme treatment of RT-PCR products before the results can be determined, and the complexity of its practical application reduces its detection efficiency (32, 33). Multiplex RT-PCR has been used for pathogen identification and detection. It involves adding multiple pairs of primers to the same reaction system, and typing strains based on the presence or length of amplified fragments (34, 35). However, this method is difficult to implement because of its high requirements for specific primer design. Recently, a multiplex real-time qRT-PCR for discriminating between vvIBDV and non-vvIBDV was developed (36), but it cannot be used for directly identifying varIBDV. In another study, a TaqMan real-time qRT-PCR was developed to distinguish varIBDV and non-varIBDV (37), but it cannot be used for directly identifying vvIBDV. Most recently, with a high-resolution melting curve qRT-PCR (HRM-qRT-PCR), the vvIBDV, varIBDV, and attIBDV can be distinguished using a reaction system (38), but this method may miss detecting other strains including cIBDV.

In this study, through the comparative analysis of massive sequences of different strains of IBDV, three specific probes with different fluorescence signals and two pairs of primers were designed to distinguish varIBDV, vvIBDV, and non- var./vvIBDV. This multiplex qRT-PCR of IBDV will have three types of result signals when the sample is positive: three fluorescent signals (FAM, VIC, and CY5) for varIBDV; two fluorescent signals (FAM and VIC) for varIBDV; one fluorescent signal (FAM) for other type of IBDV (such as cIBDV and attIBDV) but not varIBDV/vvIBDV. This multiplex qRT-PCR has good specificity and no cross reactivity with other common avian pathogens, including AIV, IBV, NDV, REV, FADV4, ARV, MDV, ALV-J/K, M. *galliseticum*, and M. *synovia*. The detection results of plasmid standards and different representative strains showed that this multiplex qRT-PCR has good repeatability and high sensitivity, with a minimum detection limit of about 10 copies. Furthermore, in laboratory or clinical sample testing, the multiplex qRT-PCR has a high consistency rate of 100% with conventional sequencing analysis methods. Among the 42 clinically positive samples tested, varIBDV, vvIBDV, and non-var/vvIBDV accounted for 64.3% (27/42), 19.0% (8/42), and 16.7% (7/42), respectively. This suggests that in 2023–2024, varIBDV and vvIBDV still are the dominant epidemic strains in China’s major poultry farming areas. Although our probe and primer designs are based on the alignment of many IBDV gene sequences from GenBank, more clinical testing, more detection alignment, and third-party validation are crucial for the maturity of the detection method.

The IBDV multiplex qRT-qPCR developed in this study demonstrates high efficiency and practicality. The entire workflow, from nucleic acid extraction to final results, requires only 2–3 h and is capable of processing up to 96 samples simultaneously in a single run. Although this multiplex qRT-PCR is developed to address the shortcomings of conventional sequencing method, it cannot replace conventional sequencing method. Under field conditions, it is rare for different strains of IBDV to infect the same bursa, but it occasionally occurs. This multiplex qRT-PCR cannot distinguish this mixed infection. In addition, as this multiplex qRT-PCR only targets viral VP2 gene, it cannot detect B-segment reassortment events of IBDV. Sanger sequencing remains necessary to detect mixed chromatogram peaks, and virus isolation is still essential for separating mixed or reassortant strains. So the IBDV multiplex qRT-qPCR is a beneficial supplement to conventional detection techniques. However, in the future, there is still room for optimization in many aspects of this multiplex qRT-qPCR. For example, changing the two-step method to a one-step method to further improve detection efficiency; further optimize the probes to detect the mix-infection of dominant strains (23); cover the probe targets with dual segments of viral genome to detect segment-reassortment of IBDV; continuously tracking the emergence of new epidemic strains to upgrade and replace existing detection system.

In summary, for the first time, this study developed a multiplex qRT-qPCR that can universally detect IBDV and simultaneously discriminate the predominant epidemic varIBDV and vvIBDV. This method is specific, sensitive, and can be used for clinical sample detection, solving the urgent need for clinical differential detection in cases of co-infection with multiple strains of IBDV.

## Data Availability

The original contributions presented in the study are included in the article/supplementary material, further inquiries can be directed to the corresponding authors.

## Footnotes

1
https://www.ncbi.nlm.nih.gov/

2
https://blast.ncbi.nlm.nih.gov/Blast.cgi
